# Bis(7-meth­oxy-1-methyl-4,9-dihydro-3*H*-β-carbolinium) tetra­chloridozincate

**DOI:** 10.1107/S1600536810010822

**Published:** 2010-03-31

**Authors:** Zukhra Ch. Kadirova, Stanislav A. Chepulsky, Nusrat A. Parpiev, Samat A. Talipov, Khasan T. Sharipov

**Affiliations:** a Tashkent Chemical-Technological Institute, Navoi St 32, Tashkent 100011, Uzbekistan; b National University of Uzbekistan, Tashkent 100123, Uzbekistan; c Institute of Biorganic Chemistry, Mirzo-Ulugbek St. 83, Tashkent 100125, Uzbekistan

## Abstract

In the title compound, (C_13_H_15_N_2_O)_2_[ZnCl_4_], also known as di(harmalinium) tetra­chloridozincate, the Zn^II^ atom is in a distorted tetrahedral coordination of the chlorido ligands. In the cation, the meth­oxy and methyl groups are both coplanar with with rings to which they are attached [maximum deviations of 0.232 (4) and 0.259 (4) Å, respectively]. In the crystal, the alkaloid cations and metal complex anions inter­act by way of N—H⋯Cl hydrogen bonds involving each Cl atom, resulting in a network structure.

## Related literature

For the activity of metal complexes with harmaline (7-methoxy-1-methyl-4,9-dihydro-3*H*-pyrido[3,4-*b*]indole), see: Al-Allaf *et al.* (1990 ▶). For the structures of harmaline and related compounds, see: Reimers *et al.* (1984 ▶); Wouters (1997 ▶); Ferretti *et al.* (2004 ▶). For zincate anions, see: Ma *et al.* (2009 ▶).
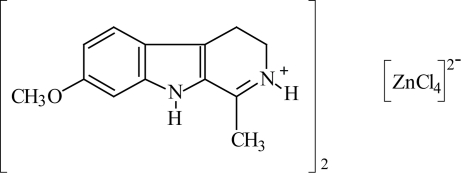


## Experimental

### 

#### Crystal data

(C_13_H_15_N_2_O)_2_[ZnCl_4_]
*M* *_r_* = 637.71
Monoclinic, 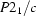

*a* = 11.2314 (2) Å
*b* = 19.1274 (2) Å
*c* = 13.5614 (2) Å
β = 107.0797 (16)°
*V* = 2784.87 (7) Å^3^
*Z* = 4
Cu *K*α radiation
μ = 5.01 mm^−1^
*T* = 293 K
0.25 × 0.12 × 0.08 mm

#### Data collection

Oxford Diffraction Xcalibur diffractometer
Absorption correction: multi-scan (*CrysAlis PRO*; Oxford Diffraction, 2007 ▶) *T*_min_ = 0.544, *T*_max_ = 0.644
12418 measured reflections
4954 independent reflections
3724 reflections with *I* > 2σ(*I*)
*R*_int_ = 0.035
3 standard reflections every 100 reflections  intensity decay: none

#### Refinement

*R*[*F* ^2^ > 2σ(*F* ^2^)] = 0.043
*wR*(*F* ^2^) = 0.116
*S* = 1.04
4954 reflections
334 parameters
H-atom parameters constrained
Δρ_max_ = 0.54 e Å^−3^
Δρ_min_ = −0.46 e Å^−3^

### 

Data collection: *CrysAlis PRO* (Oxford Diffraction, 2007 ▶); cell refinement: *CrysAlis PRO*; data reduction: *CrysAlis PRO*; program(s) used to solve structure: *SHELXS97* (Sheldrick, 2008 ▶); program(s) used to refine structure: *SHELXL97* (Sheldrick, 2008 ▶); molecular graphics: *ORTEP-3 for Windows* (Farrugia, 1997 ▶); software used to prepare material for publication: *publCIF* (Westrip, 2010 ▶).

## Supplementary Material

Crystal structure: contains datablocks I, global. DOI: 10.1107/S1600536810010822/bv2139sup1.cif

Structure factors: contains datablocks I. DOI: 10.1107/S1600536810010822/bv2139Isup2.hkl

Additional supplementary materials:  crystallographic information; 3D view; checkCIF report

## Figures and Tables

**Table 1:** Hydrogen-bond geometry (Å, °)

*D*—H⋯*A*	*D*—H	H⋯*A*	*D*⋯*A*	*D*—H⋯*A*
N1—H1*A*⋯Cl2^i^	0.86	2.45	3.307 (3)	171
N2—H2*A*⋯Cl1^ii^	0.86	2.46	3.299 (3)	166
N3—H3*C*⋯Cl3	0.86	2.33	3.183 (3)	173
N4—H4*C*⋯Cl4^iii^	0.86	2.44	3.287 (3)	170

Symmetry codes: (i) 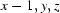; (ii) 
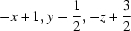
; (iii) 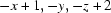.

**Fig. 1.**
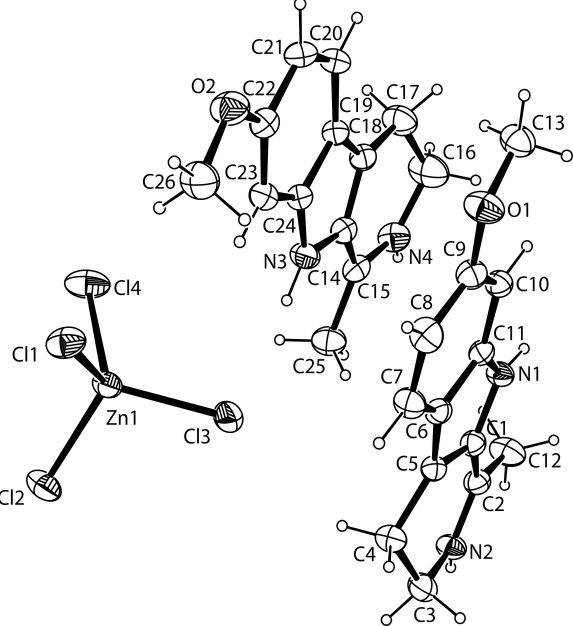
A view of the structure of (I), showing the atom numbering scheme. Displacement ellipsoids are drawn at the 50% probability level and H atoms are shown as small spheres of arbitary radii.

**Fig. 2.**
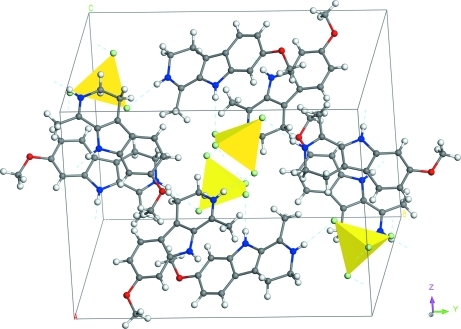
The crystal structure packing scheme showing the hydrogen bonds system.

## supplementary crystallographic information

### Comment

The metal complexes of the harmaline (7-methoxy-1-methyl-4,9-dihydro-3*H*-β-carboline) and other carboline alkaloids have biological activity (Al-Allaf *et al.* 1990). In this study we synthesized the molecular salt containing a zinc-chlorido complex and harmalinium cations, and report the structure of the title compound, (I).

The molecular structure is shown on Fig.1 and geometrical parameters are available from archived CIF.

Zinc ions are anions [ZnCl_4_]^2-^ have distorted tetragonal configuration (Sutherland *et al.*, 2009) and the valence angles are close to tetrahedral, being in the ranges 104.15 (4)-113.37 (4)°. The Zn—Cl bonds are not equal and values of bond lengths, being in the range 2.2536 (10)-2.2885 (10)Å .

Bond lengths and angles in the cations do not differ from their normal values. The alkaloid molecules are in a protonated form and the proton is localized at the nitrogen atom in the carboline ring as observed previously in structure of harmaline hydrochloride (Ferretti *et al.*, 2004; Wouters, 1997). Protonization leads to a decrease in CH_2_— CH_2_ and C═N bond lengths in comparison with the harmaline structure (Reimers *et al.*, 1984). In the pyrrole cycle C—NH bond lengths are not equivalent as distinctions are expressed in a greater degree than for the harmaline crystal structure (Reimers *et al.*, 1984). Both methoxy and methyl groups are located in the plane of the pyrrole and benzole rings and the carboline ring has a noncoplanar conformation with the torsion angles shown in the table 1. The sp^3^-hybridizied carbon atoms in the cycle being displaced from the mean plane of carboline cycle by -0.286 (4), - 0.199 (4), 0.257 (5) and -0.090 (4) Å for C3, C4, C16, C17, respectively.

Unlike the parent harmaline structure (Reimers *et al.*, 1984) in which only one hydrogen bond is present, in case of compound I a network of hydrogen bonds is formed (Fig.2) as in case of harmine hydrochloride where hydrogen bonds are formed between NH-groups as acceptors and chlorine atoms as donors of electrons (Ferretti *et al., *2004; Wouters, 1997).

### Experimental

The ZnCl_2 _(1 mmol) and harmaline (2.5 mmol) were heated on water bath in 2M solution of hydrochloric acid in ethanol. The resulting solution yielded colourless crystals which were filtered off and washed twice with acetone. Elem. Analysis found: C 49.0, H 4.7, N 8.8, Zn 10.3%; requires: C 49.0, H 4.7, N 8.8, Zn 10.3%. Crystals of the title compound, suitable to X-ray diffraction analysis, were selected directly from the sample as prepared.

### Refinement

All e.s.d.'s (except the e.s.d. in the dihedral angle between two l.s. planes) are estimated using the full covariance matrix. The cell e.s.d.'s are taken into account individually in the estimation of e.s.d.'s in distances, angles and torsion angles; correlations between e.s.d.'s in cell parameters are only used when they are defined by crystal symmetry. An approximate (isotropic) treatment of cell e.s.d.'s is used for estimating e.s.d.'s involving l.s. planes.

Refinement of F^2^ against ALL reflections. The weighted R-factor wR and goodness of fit S are based on F^2^, conventional R-factors R are based on F, with F set to zero for negative F^2^. The threshold expression of F^2^ > 2sigma(F^2^) is used only for calculating R-factors(gt) etc. and is not relevant to the choice of reflections for refinement. R-factors based on F^2^ are statistically about twice as large as those based on F, and R- factors based on ALL data will be even larger.

All the H-atoms were included in calculated positions [N—H = 0.88 Å, C—H = 0.93 - 0.96 Å] and treated as riding atoms [U_iso_(H) = k × U_eq_(parent atom], where k = 1.2 for NH_2_ and CH H atoms and 1.5 for methyl H atomshydrogens].

### Crystal data

**Table:**

(C_13_H_15_N_2_O)_2_[ZnCl_4_]	*F*(000) = 1312
*M**_r_* = 637.71	*D*_x_ = 1.521 Mg m^−^^3^
Monoclinic, *P*2_1_/*c*	Cu *K*α radiation, λ = 1.54184 Å
*a* = 11.2314 (2) Å	Cell parameters from 4954 reflections
*b* = 19.1274 (2) Å	θ = 4.1–71.0°
*c* = 13.5614 (2) Å	µ = 5.01 mm^−^^1^
β = 107.0797 (16)°	*T* = 293 K
*V* = 2784.87 (7) Å^3^	Monoclinic, colourless
*Z* = 4	0.25 × 0.12 × 0.08 mm

### Data collection

**Table:**

Oxford Diffraction Xcalibur diffractometer	3724 reflections with *I* > 2σ(*I*)
Radiation source: fine-focus sealed tube	*R*_int_ = 0.035
graphite	θ_max_ = 71.0°, θ_min_ = 4.1°
heavy atom scans	*h* = −11→13
Absorption correction: multi-scan (*CrysAlis PRO*; Oxford Diffraction, 2007)	*k* = −18→22
*T*_min_ = 0.544, *T*_max_ = 0.644	*l* = −16→16
12418 measured reflections	3 standard reflections every 120 reflections
4954 independent reflections	intensity decay: none

### Refinement

**Table:**

Refinement on *F*^2^	Primary atom site location: structure-invariant direct methods
Least-squares matrix: full	Secondary atom site location: difference Fourier map
*R*[*F*^2^ > 2σ(*F*^2^)] = 0.043	Hydrogen site location: inferred from neighbouring sites
*wR*(*F*^2^) = 0.116	H-atom parameters constrained
*S* = 1.04	*w* = 1/[σ^2^(*F*_o_^2^) + (0.0588*P*)^2^ + 1.2361*P*] where *P* = (*F*_o_^2^ + 2*F*_c_^2^)/3
4954 reflections	(Δ/σ)_max_ < 0.001
334 parameters	Δρ_max_ = 0.54 e Å^−^^3^
0 restraints	Δρ_min_ = −0.46 e Å^−^^3^
0 constraints	

### Special details

**Table:**

Geometry. All esds (except the esd in the dihedral angle between two l.s. planes) are estimated using the full covariance matrix. The cell esds are taken into account individually in the estimation of esds in distances, angles and torsion angles; correlations between esds in cell parameters are only used when they are defined by crystal symmetry. An approximate (isotropic) treatment of cell esds is used for estimating esds involving l.s. planes.
Refinement. Refinement of *F*^2^ against ALL reflections. The weighted *R*-factor wR and goodness of fit *S* are based on *F*^2^, conventional *R*-factors *R* are based on *F*, with *F* set to zero for negative *F*^2^. The threshold expression of *F*^2^ > σ(*F*^2^) is used only for calculating *R*-factors(gt) etc. and is not relevant to the choice of reflections for refinement. *R*-factors based on *F*^2^ are statistically about twice as large as those based on *F*, and *R*- factors based on ALL data will be even larger.

### Fractional atomic coordinates and isotropic or equivalent isotropic displacement parameters (Å^2^)

**Table:**

	*x*	*y*	*z*	*U*_iso_*/*U*_eq_	
Zn1	0.76391 (4)	0.05281 (2)	0.78045 (4)	0.04473 (15)	
Cl1	0.80185 (10)	0.14137 (5)	0.68163 (7)	0.0583 (3)	
Cl2	0.90754 (9)	−0.03331 (5)	0.80919 (8)	0.0623 (3)	
Cl3	0.57608 (8)	0.00479 (5)	0.70451 (9)	0.0678 (3)	
Cl4	0.77170 (12)	0.10536 (5)	0.93383 (8)	0.0734 (3)	
O1	0.1080 (2)	0.18482 (13)	0.5051 (2)	0.0595 (7)	
N1	0.0972 (2)	−0.04362 (13)	0.6637 (2)	0.0402 (6)	
H1A	0.0414	−0.0400	0.6953	0.048*	
N2	0.2328 (3)	−0.21801 (15)	0.6886 (2)	0.0480 (7)	
H2A	0.2367	−0.2566	0.7221	0.058*	
C1	0.1660 (3)	−0.10271 (17)	0.6593 (2)	0.0400 (7)	
C2	0.1632 (3)	−0.16743 (17)	0.7074 (3)	0.0444 (8)	
C3	0.3039 (3)	−0.21253 (19)	0.6142 (3)	0.0540 (9)	
H3A	0.2531	−0.2301	0.5482	0.065*	
H3B	0.3768	−0.2423	0.6369	0.065*	
C4	0.3451 (3)	−0.14021 (19)	0.5997 (3)	0.0508 (9)	
H4A	0.4222	−0.1300	0.6529	0.061*	
H4B	0.3613	−0.1370	0.5334	0.061*	
C5	0.2495 (3)	−0.08782 (17)	0.6044 (2)	0.0390 (7)	
C6	0.2285 (3)	−0.01784 (17)	0.5711 (2)	0.0388 (7)	
C7	0.2806 (3)	0.02628 (19)	0.5116 (2)	0.0476 (8)	
H7A	0.3440	0.0104	0.4860	0.057*	
C8	0.2367 (3)	0.0920 (2)	0.4926 (3)	0.0521 (9)	
H8A	0.2696	0.1214	0.4526	0.063*	
C9	0.1411 (3)	0.11741 (18)	0.5325 (2)	0.0451 (8)	
C10	0.0876 (3)	0.07649 (17)	0.5905 (2)	0.0402 (7)	
H10A	0.0247	0.0932	0.6161	0.048*	
C11	0.1329 (3)	0.00793 (16)	0.6094 (2)	0.0357 (7)	
C12	0.0871 (4)	−0.1803 (2)	0.7790 (3)	0.0640 (11)	
H12A	0.0985	−0.2277	0.8032	0.096*	
H12B	0.0007	−0.1725	0.7433	0.096*	
H12C	0.1130	−0.1490	0.8367	0.096*	
C13	0.0274 (4)	0.2179 (2)	0.5539 (3)	0.0582 (10)	
H13A	0.0111	0.2649	0.5286	0.087*	
H13B	0.0661	0.2188	0.6271	0.087*	
H13C	−0.0495	0.1925	0.5389	0.087*	
N3	0.3936 (3)	0.10991 (15)	0.7707 (2)	0.0473 (7)	
H3C	0.4395	0.0784	0.7550	0.057*	
N4	0.2175 (3)	0.03927 (17)	0.9329 (2)	0.0573 (8)	
H4C	0.2128	0.0035	0.9700	0.069*	
O2	0.4732 (3)	0.32178 (13)	0.5990 (2)	0.0626 (7)	
C14	0.3153 (3)	0.10024 (18)	0.8312 (2)	0.0437 (8)	
C15	0.3010 (3)	0.03874 (19)	0.8826 (3)	0.0482 (8)	
C16	0.1328 (5)	0.0970 (2)	0.9292 (4)	0.0863 (15)	
H16A	0.0549	0.0866	0.8771	0.104*	
H16B	0.1153	0.0994	0.9950	0.104*	
C17	0.1742 (4)	0.1655 (2)	0.9072 (3)	0.0649 (11)	
H17A	0.2174	0.1882	0.9717	0.078*	
H17B	0.1019	0.1937	0.8734	0.078*	
C18	0.2582 (3)	0.16256 (18)	0.8406 (2)	0.0442 (8)	
C19	0.3027 (3)	0.21319 (18)	0.7847 (2)	0.0422 (7)	
C20	0.2842 (3)	0.28532 (19)	0.7657 (3)	0.0495 (8)	
H20A	0.2319	0.3102	0.7949	0.059*	
C21	0.3427 (3)	0.31842 (18)	0.7049 (3)	0.0508 (9)	
H21A	0.3302	0.3661	0.6926	0.061*	
C22	0.4230 (3)	0.28139 (18)	0.6597 (3)	0.0474 (8)	
C23	0.4460 (3)	0.21113 (18)	0.6765 (3)	0.0462 (8)	
H23A	0.4988	0.1870	0.6470	0.055*	
C24	0.3857 (3)	0.17796 (17)	0.7401 (2)	0.0418 (7)	
C25	0.3748 (4)	−0.0254 (2)	0.8814 (3)	0.0622 (10)	
H25A	0.3504	−0.0613	0.9209	0.093*	
H25B	0.4618	−0.0154	0.9108	0.093*	
H25C	0.3601	−0.0410	0.8115	0.093*	
C26	0.5400 (4)	0.2872 (2)	0.5392 (3)	0.0639 (11)	
H26A	0.5706	0.3211	0.5003	0.096*	
H26B	0.4858	0.2550	0.4929	0.096*	
H26C	0.6090	0.2622	0.5841	0.096*	

### Atomic displacement parameters (Å^2^)

**Table:**

	*U*^11^	*U*^22^	*U*^33^	*U*^12^	*U*^13^	*U*^23^
Zn1	0.0481 (3)	0.0351 (2)	0.0585 (3)	0.0031 (2)	0.0273 (2)	0.0015 (2)
Cl1	0.0841 (6)	0.0390 (5)	0.0623 (5)	−0.0064 (4)	0.0377 (5)	0.0017 (4)
Cl2	0.0606 (5)	0.0537 (5)	0.0827 (6)	0.0188 (4)	0.0365 (5)	0.0093 (5)
Cl3	0.0475 (5)	0.0542 (6)	0.1095 (8)	−0.0051 (4)	0.0350 (5)	−0.0148 (6)
Cl4	0.1199 (9)	0.0529 (6)	0.0611 (5)	0.0163 (6)	0.0481 (6)	0.0022 (5)
O1	0.0744 (17)	0.0393 (14)	0.0765 (17)	0.0082 (13)	0.0404 (15)	0.0158 (13)
N1	0.0428 (14)	0.0343 (15)	0.0504 (15)	−0.0003 (12)	0.0241 (12)	0.0002 (12)
N2	0.0563 (17)	0.0316 (15)	0.0590 (16)	0.0076 (13)	0.0214 (14)	0.0037 (13)
C1	0.0412 (17)	0.0359 (17)	0.0420 (16)	−0.0014 (14)	0.0111 (14)	0.0004 (14)
C2	0.0469 (18)	0.0370 (18)	0.0516 (19)	−0.0035 (16)	0.0179 (15)	−0.0037 (15)
C3	0.050 (2)	0.048 (2)	0.065 (2)	0.0071 (17)	0.0193 (18)	−0.0009 (19)
C4	0.056 (2)	0.046 (2)	0.055 (2)	0.0059 (17)	0.0230 (17)	−0.0025 (17)
C5	0.0387 (16)	0.0397 (18)	0.0395 (16)	−0.0007 (14)	0.0129 (14)	−0.0052 (14)
C6	0.0418 (17)	0.0381 (18)	0.0365 (15)	−0.0017 (14)	0.0117 (13)	−0.0052 (14)
C7	0.0515 (19)	0.051 (2)	0.0470 (18)	0.0020 (17)	0.0254 (16)	0.0018 (17)
C8	0.056 (2)	0.054 (2)	0.0528 (19)	−0.0036 (18)	0.0251 (17)	0.0096 (18)
C9	0.052 (2)	0.0370 (18)	0.0459 (17)	−0.0005 (16)	0.0145 (15)	0.0049 (15)
C10	0.0418 (17)	0.0360 (17)	0.0445 (17)	−0.0007 (14)	0.0154 (14)	0.0003 (14)
C11	0.0390 (16)	0.0323 (16)	0.0364 (15)	−0.0045 (13)	0.0121 (13)	−0.0008 (13)
C12	0.079 (3)	0.041 (2)	0.087 (3)	0.007 (2)	0.048 (2)	0.015 (2)
C13	0.060 (2)	0.046 (2)	0.071 (2)	0.0085 (18)	0.0224 (19)	0.0100 (19)
N3	0.0495 (16)	0.0385 (16)	0.0592 (16)	0.0045 (13)	0.0243 (14)	−0.0017 (14)
N4	0.070 (2)	0.0500 (19)	0.0582 (17)	−0.0025 (16)	0.0281 (16)	0.0031 (15)
O2	0.0742 (17)	0.0449 (15)	0.0789 (17)	−0.0053 (13)	0.0384 (15)	−0.0002 (14)
C14	0.0442 (18)	0.0416 (19)	0.0452 (17)	−0.0028 (15)	0.0127 (15)	−0.0054 (15)
C15	0.053 (2)	0.045 (2)	0.0451 (18)	−0.0054 (16)	0.0107 (16)	−0.0062 (16)
C16	0.096 (3)	0.070 (3)	0.119 (4)	0.004 (3)	0.071 (3)	0.008 (3)
C17	0.072 (3)	0.061 (3)	0.075 (3)	0.007 (2)	0.041 (2)	−0.001 (2)
C18	0.0444 (18)	0.046 (2)	0.0421 (17)	−0.0009 (16)	0.0124 (15)	−0.0087 (15)
C19	0.0414 (17)	0.0404 (18)	0.0440 (17)	0.0019 (14)	0.0114 (14)	−0.0082 (15)
C20	0.051 (2)	0.0395 (19)	0.060 (2)	0.0088 (16)	0.0193 (17)	−0.0090 (17)
C21	0.059 (2)	0.0306 (17)	0.063 (2)	0.0016 (16)	0.0180 (18)	−0.0051 (17)
C22	0.0457 (18)	0.0406 (19)	0.0560 (19)	−0.0041 (16)	0.0155 (16)	−0.0038 (16)
C23	0.0437 (18)	0.0421 (19)	0.0550 (19)	0.0021 (16)	0.0179 (16)	−0.0051 (16)
C24	0.0388 (17)	0.0348 (18)	0.0495 (18)	0.0004 (14)	0.0094 (14)	−0.0061 (15)
C25	0.077 (3)	0.045 (2)	0.068 (2)	0.006 (2)	0.026 (2)	0.0060 (19)
C26	0.069 (3)	0.061 (3)	0.072 (2)	−0.002 (2)	0.037 (2)	0.001 (2)

### Geometric parameters (Å, °)

**Table:**

Zn1—Cl3	2.2536 (10)	C14—C18	1.377 (5)
Zn1—Cl2	2.2580 (10)	C14—C15	1.400 (5)
Zn1—Cl1	2.2765 (9)	C15—C25	1.484 (5)
Zn1—Cl4	2.2885 (10)	C16—C17	1.449 (6)
O1—C9	1.363 (4)	C17—C18	1.487 (5)
O1—C13	1.417 (4)	C18—C19	1.409 (5)
N1—C11	1.359 (4)	C19—C20	1.407 (5)
N1—C1	1.380 (4)	C19—C24	1.422 (4)
N2—C2	1.315 (4)	C20—C21	1.353 (5)
N2—C3	1.463 (4)	C21—C22	1.420 (5)
C1—C5	1.388 (4)	C22—C23	1.375 (5)
C1—C2	1.404 (4)	C23—C24	1.396 (5)
C2—C12	1.491 (5)	N1—H1A	0.86
C3—C4	1.490 (5)	N2—H2A	0.86
C4—C5	1.484 (4)	C3—H3A	0.97
C5—C6	1.410 (5)	C3—H3B	0.97
C6—C7	1.407 (4)	C4—H4A	0.97
C6—C11	1.412 (4)	C4—H4B	0.97
C7—C8	1.348 (5)	C7—H7A	0.93
C8—C9	1.422 (5)	C8—H8A	0.93
C9—C10	1.367 (4)	C10—H10A	0.93
C10—C11	1.403 (4)	C12—H12A	0.96
N3—C24	1.361 (4)	C12—H12B	0.96
N3—C14	1.381 (4)	C12—H12C	0.96
N4—C15	1.312 (5)	C13—H13A	0.96
N4—C16	1.450 (5)	C13—H13B	0.96
O2—C22	1.365 (4)	C13—H13C	0.96
O2—C26	1.420 (4)		
			
Cl3—Zn1—Cl2	107.88 (4)	C19—C18—C17	133.6 (3)
Cl3—Zn1—Cl1	110.26 (4)	C20—C19—C18	135.7 (3)
Cl2—Zn1—Cl1	113.37 (4)	C20—C19—C24	117.8 (3)
Cl3—Zn1—Cl4	112.03 (4)	C18—C19—C24	106.5 (3)
Cl2—Zn1—Cl4	109.20 (4)	C21—C20—C19	119.9 (3)
Cl1—Zn1—Cl4	104.15 (4)	C20—C21—C22	120.9 (3)
C9—O1—C13	117.1 (3)	O2—C22—C23	124.2 (3)
C11—N1—C1	108.2 (2)	O2—C22—C21	113.9 (3)
C2—N2—C3	123.8 (3)	C23—C22—C21	121.9 (3)
N1—C1—C5	109.3 (3)	C22—C23—C24	116.4 (3)
N1—C1—C2	127.8 (3)	N3—C24—C23	128.5 (3)
C5—C1—C2	122.8 (3)	N3—C24—C19	108.4 (3)
N2—C2—C1	117.6 (3)	C23—C24—C19	123.1 (3)
N2—C2—C12	119.5 (3)	C1—N1—H1A	126
C1—C2—C12	122.9 (3)	C11—N1—H1A	126
N2—C3—C4	114.3 (3)	C2—N2—H2A	118
C5—C4—C3	111.4 (3)	C3—N2—H2A	118
C1—C5—C6	106.9 (3)	N2—C3—H3A	109
C1—C5—C4	119.7 (3)	N2—C3—H3B	109
C6—C5—C4	133.0 (3)	C4—C3—H3A	109
C7—C6—C5	134.0 (3)	C4—C3—H3B	109
C7—C6—C11	119.4 (3)	H3A—C3—H3B	108
C5—C6—C11	106.7 (3)	C3—C4—H4A	109
C8—C7—C6	118.6 (3)	C3—C4—H4B	109
C7—C8—C9	121.4 (3)	C5—C4—H4A	109
O1—C9—C10	124.6 (3)	C5—C4—H4B	109
O1—C9—C8	113.3 (3)	H4A—C4—H4B	108
C10—C9—C8	122.1 (3)	C6—C7—H7A	121
C9—C10—C11	116.3 (3)	C8—C7—H7A	121
N1—C11—C10	128.9 (3)	C7—C8—H8A	119
N1—C11—C6	108.8 (3)	C9—C8—H8A	119
C10—C11—C6	122.2 (3)	C9—C10—H10A	122
C24—N3—C14	108.3 (3)	C11—C10—H10A	122
C15—N4—C16	123.4 (3)	C2—C12—H12A	110
C22—O2—C26	117.5 (3)	C2—C12—H12B	109
C18—C14—N3	109.5 (3)	C2—C12—H12C	109
C18—C14—C15	123.8 (3)	H12A—C12—H12B	109
N3—C14—C15	126.5 (3)	H12A—C12—H12C	109
N4—C15—C14	117.1 (3)	H12B—C12—H12C	109
N4—C15—C25	120.0 (3)	O1—C13—H13A	109
C14—C15—C25	122.9 (3)	O1—C13—H13B	109
C17—C16—N4	116.7 (4)	O1—C13—H13C	109
C16—C17—C18	113.0 (3)	H13A—C13—H13B	109
C14—C18—C19	107.3 (3)	H13A—C13—H13C	109
C14—C18—C17	119.0 (3)	H13B—C13—H13C	110
			
C13—O1—C9—C8	−170.7 (3)	C3—C4—C5—C6	−161.5 (3)
C13—O1—C9—C10	10.7 (4)	C1—C5—C6—C7	−178.5 (3)
C11—N1—C1—C2	177.5 (3)	C1—C5—C6—C11	1.1 (3)
C11—N1—C1—C5	2.0 (3)	C4—C5—C6—C7	8.2 (6)
C1—N1—C11—C6	−1.3 (3)	C4—C5—C6—C11	−172.2 (3)
C1—N1—C11—C10	178.6 (3)	C5—C6—C7—C8	179.2 (3)
C3—N2—C2—C1	−5.5 (5)	C11—C6—C7—C8	−0.4 (4)
C3—N2—C2—C12	175.5 (3)	C5—C6—C11—N1	0.1 (3)
C2—N2—C3—C4	29.2 (5)	C5—C6—C11—C10	−179.83
N1—C1—C2—N2	176.8 (3)	C7—C6—C11—N1	179.8 (3)
N1—C1—C2—C12	−4.2 (6)	C7—C6—C11—C10	−0.2 (4)
C5—C1—C2—N2	−8.3 (5)	C6—C7—C8—C9	0.9 (5)
C5—C1—C2—C12	170.7 (3)	C7—C8—C9—O1	−179.7 (3)
N1—C1—C5—C4	172.5 (3)	C7—C8—C9—C10	−1.1 (5)
N1—C1—C5—C6	−1.9 (3)	O1—C9—C10—C11	179.0 (3)
C2—C1—C5—C4	−3.3 (5)	C8—C9—C10—C11	0.5 (4)
C2—C1—C5—C6	−177.7 (3)	C9—C10—C11—N1	−179.8 (3)
N2—C3—C4—C5	−36.8 (4)	C9—C10—C11—C6	0.1 (4)
C3—C4—C5—C1	25.9 (4)		

### Hydrogen-bond geometry (Å, °)

**Table:**

*D*—H···*A*	*D*—H	H···*A*	*D*···*A*	*D*—H···*A*
N1—H1A···Cl2^i^	0.86	2.45	3.307 (3)	171
N2—H2A···Cl1^ii^	0.86	2.46	3.299 (3)	166
N3—H3C···Cl3	0.86	2.33	3.183 (3)	173
N4—H4C···Cl4^iii^	0.86	2.44	3.287 (3)	170

Symmetry codes: (i) *x*−1, *y*, *z*; (ii) −*x*+1, *y*−1/2, −*z*+3/2; (iii) −*x*+1, −*y*, −*z*+2.
